# Circumstances and consequences of falls in elderly people with vestibular disorder

**DOI:** 10.1016/S1808-8694(15)30974-5

**Published:** 2015-10-19

**Authors:** Fernando Freitas Ganança, Juliana Maria Gazzola, Mayra Cristina Aratani, Monica Rodrigues Perracini, Maurício Malavasi Ganança

**Affiliations:** a Physician, ENT specialist, Doctor in Medicine at UNIFESP - EPM. Affiliated Professor of Otoneurology at UNIFESP - EPM. Professor of the post-graduate course on Neuromotor Rehabilitation Science at UNIBAN; b Physical therapist. Specialist in Gerontology, graduated at UNIFESP - EPM. Master in Sciences, graduate from the post-graduate course in Otorhinolaryngology and Head & Neck Surgery at UNIFESP - EPM; c Physical therapist. Specialist in Gerontology, graduated at UNIFESP - EPM; d Physical therapist. Doctor in Rehabilitation Science - UNIFESP - EPM. Coordinating Professor of the Physical Therapy master's degree course at UNICID; e ^5^ Full Professor Otorhinolaryngology at UNIFESP - EPM. Senior Researcher in the stricto sensu Post-Graduate Program (Master's degree) on Neuromotor Rehabilitation Science at UNIBAN. Responsible for the Balance Metrics Unit of the Otorhinolaryngology Sector of the Fleury Diagnostic Medicine Center - Sao Paulo (SP). Otoneurology Department at UNIFESP - EPM / Vestibular Rehabilitation Sector

**Keywords:** vestibular disease, elderly, falls, dizziness

## Abstract

**Aim:**

To investigate the circumstances and consequences of falls in the chronically dizzy elderly and to correlate them with the number of falls (one/two and more).

**Method:**

Transversal descriptive analytic study with 64 patients aged 65 or over, with history of falls and diagnostic of chronic vestibular dysfunction. We performed a descriptive analysis and Qui-Square test (χ<0.05).

**Results:**

The sample was constituted by a female majority (76.6%) with a mean age of 73.62±5.69 years. The vestibular examination showed peripheral vestibulopathy in 81.5% of the cases and the most prevalent diagnostic hypothesis were benign paroxysmal positional vertigo (43.8%) and metabolic inner ear disease (42.2%). Recurrent falls were seen in 35 elderly (53.1%). In relation to the last fall, 39.1% of the patients had fallen in their homes, 51.6% of them occurred during the morning, 51.6% with some propulsion mechanism, 53.1% when walking, 25.0% caused by dizziness and 23.4% by stumbling. Activity restriction was significantly greater in patients that have already had two and more falls, when compared with those who had fallen only once (p=0.031). We found a significant association between the number of falls and their causes (p<0.001). Falls that have happened by slipping were more frequent in the elderly that reported one fall (p=0.0265) and falls that had happened because of dizziness were more frequent in the elderly that complained of two or more falls (p=0.0012).

**Conclusions:**

Fear and tendency to fall are referred by the majority of chronically dizzy elderly. Fall are more frequent in the morning, in the home and during walking. The propulsion direction is mentioned by half of the elderly and the most common cause for falls are dizziness and stumbling. The number of falls is significantly associated with activity restrictions after the last fall and with the causes for falling (slipping and dizziness).

## INTRODUCTION

Dizziness is a sensation of loss of body balance. It may be defined as erroneous perception, a movement illusion or hallucination, a sensation of rotating spatial disorientation (vertigo) or non-rotating spatial disorientation (instability, imbalance, fluctuation, oscillation, oscillopsy). Different types of dizziness (rotatory and non-rotatory) in the same individual are not rare. Both types may or may not be vestibular disorders that can be demonstrated by otoneurological tests^1^. Dizziness results from primary or secondary functional disorders of the vestibular system in approximately 85% of cases^2^.

Falls are the main complication of balance disorders in the elderly. A fall is defined as an unintentional event resulting in a change of position to a lower level compared to the individual's initial position, with no intrinsic determining factor such as a cerebral vascular accident or syncope or an unavoidable accident^3^, ^4^.

In study done in metropolitan Sao Paulo, Perracini and Ramos^5^ found that the prevalence of falls among elderly people was about 30% and that the rate of recurrent falls was 11% in that group. Although most falls (30 to 50%) cause minor injuries that do not require medical care, 10 to 15% of falls cause serious injuries, the most common being hip fracture (1% to 2%). Approximately 25% of falls in that community cause immediate limitation of activities either due to physical harm or to fear of falling^4^.

In a study on the incidence of falls in individuals with vestibular dysfunction, those with bilateral vestibular deficits fell more often compared to patients with unilateral involvement (respectively 51.1% and 30%). Patients with unilateral vestibular deficits aged over 75 years had a higher rate of falls compared to patients aged below 65 years. However, in patients with bilateral vestibular deficits, the rate of falls in patients aged 75 years and above was lower compared to patients aged less than 74 years, probably due to increased demands to use attention and a reduction of activities, with less exposure to situations of risk. No difference was found in the incidence of falls in patients with unilateral vestibular dysfunction and patients aged 65 years or more. There was a significantly higher number of falls in patients with bilateral vestibular dysfunction in the 65 to 74 years age group compared to the elderly in the community within the same age group^6^.

Falls frequently result from a sum of intrinsic and extrinsic risk factors; it is difficult to restrict a fall to a single risk factor or cause. Fall-related intrinsic factors in the elderly are loss of mobility, functional incapacity to undertake activities of daily life, reduced lower limb muscle strength, balance disorders, complaints of dizziness, psychotropic medication, visual, auditory and/or cognitive deficits, postural hypotension, gait disorders and chronic diseases. Extrinsic factors are environmental risks (poor lighting, slippery floors and others), risky behaviors (climbing chairs) and the specific task in a given moment.7 When demands on postural control are greater that the individual capacity to deal with them, there is a fall^8^.

No studies on the circumstances and consequences of falls in the elderly with chronic vestibular diseases were found in national and international literature. Identifying these characteristics may contribute to establish specific prevention strategies to reduce the rate of falls and its complications.

The aim of this study was to investigate the circumstances and consequences of falls in the elderly with chronic vestibular diseases, relating them to the number of falls (one/two or more falls).

## METHODS

An analytical descriptive cross-sectional study approved by the Federal University of Sao Paulo - Sao Paulo Medical School (UNIFESP - EPM) Research Ethics Committee, protocol number 01214/05. This paper is part of a study financed by the Sao Paulo State Research Support Foundation (FAPESP), process number 03/10119-3.

The sample included male and female patients from the UNIFESP - EPM Otoneurology Outpatient Clinic aged 65 years and over, with chronic vestibular disease, characterized by complaints of dizziness and/or imbalance and/or dazing and/or other non-specific sensations of dizziness with at least three months duration. The evaluation was not made during a crisis of vertigo.

Exclusion criteria included elderly patients with poor or incapacitating visual and auditory acuity which limited activities of daily life even with corrective lenses and/or hearing aids, those that had undergone upper and/or lower limb amputation and patients that could not walk independently or were confined to wheelchairs.

Patients initially underwent otoneurological clinical evaluation, which includes the clinical history, the otorhinolaryngological physical examination, audiometry, imitanciometry and vestibular examination using vectoelectronystagmography according to criteria described in literature^9^, ^10^.

Patients were inquired about falls during the year preceding the study. Information was collected about the place, time, lighting, direction, causes and activities at the moment of the fall; information was also gathered about whether the fall led to restrictions on activities of daily life. The last event was investigated when there were recurrent falls (two or more falls). Patients were also inquired about the tendency to fall and fear of falls.

Statistical analysis was descriptive analysis. For the analysis of inference, the variable “number of falls” was compared with the variables place, time, lighting, direction and causes of the fall, activities at the moment of the fall and if the fall led to restrictions on activities of daily life; the Chi-squared test was used to calculate these variables (c2). Fisher's accuracy test was used to identify the association between variables of contingency tables where 25% of cells had less than five occurrences. The Chi-squared test was used to analyze the association between frequencies of a sample with two categories, followed by Yates’ correction. The statistical test significance level was 5% (a ≤ 0,05).

Analysis was done using the software SPSS 10.0 under Windows^11^.

## RESULTS

Our sample included 64 elderly patients with a diagnosis of chronic vestibular syndrome monitored in our outpatient department. Average age was 73.62 years, standard deviation (SD) of 5.69; the maximum age was 89 years. Fifteen (23.4%) were male and 49 (76.6%) were female.

The average number of diseases per individual was 3.98 (SD = 1.77), with a maximum of eight associated diseases. The most prevalent were circulatory system diseases (78.1%) followed by osteomuscular system and connective tissue diseases (67.2%), and nutritional endocrine and metabolic diseases (54.7%). The average number of medical drugs used was 4.08 drugs (SD = 2.38), with maximum of ten drugs.

Results of the vestibular test showed peripheral syndrome (81.5%), no abnormalities (11.1%) and central vestibular syndrome (7.4%). The most frequent vestibular disorders were benign paroxysmal positional vertigo (43.8%) and metabolic diseases of the labyrinth (42.2%).

Seven patients (10.9%) used walking support devices. Of the 64 elderly patients, 34 (53.1%) had reported recurrent falls. Fear of falls and the tendency to fall were reported by most patients (73.4% and 82.8%, respectively).

The most common time for a fall was the morning (51.6%), followed by the afternoon (34.4%), the evening (12.5%) and dawn (1.6%).

The reported direction of the fall was propulsion in 51.6% of the patients, retropulsion in 15.6%, right lateral in 15.6%, left lateral in 12.5% and no answer in 4.7%.

The fall places are described on Table 1. Falls in the bathroom were 38.1% of fall that occurred at home. In most cases the house was well lighted (85.9%).

**Table 1:** Absolute and relative frequency of fall places in elderly patients with chronic vestibular dysfunction (n = 64).

Place of the fall	Absolute Frequency (n)	Relative Frequency (%)
At home (external environment)	13	20,3
At home (internal environment)	21	32,8
Out of home (familiar place)	25	39,1
Out of home (unfamiliar place)	5	7,8

The most frequent tasks at the moment of the fall were walking (53.1%), climbing up or down stairs (10.9%), change of posture (9.4%) and taking a bath (6.3%).

Vertigo was most common cause of falls (25.0%), followed by tripping over (23.4%), slipping (20.3%), syncope/blackout (10.8%) and others (20.5%). “Other” causes include reduced attention at the moment of the fall (7.9%), weakening of the knees (7.9%) and an unexpected hurdle (4.7%).

Fourteen patients (21.9%) reported restriction of activities of daily life after the last fall.

There was a significant association between number of falls and restriction of activities after the last fall (p=0.031). Restriction of activities was significantly higher in elderly patients with vestibular diseases that had had two or more falls (78.6%), compared to those with only one fall (21.4%).

A significant association was found between the number and the causes of falls (p < 0.001). There was a statistically significant difference between the number of falls and slipping (p=0,026), among other causes of falls; there was an increased rate of slipping in patients that reported falls. Falls due to dizziness or vertigo were more frequent in patients that reported two or more falls during the study period, which was statistically significant (p=0.001) as seen on Table 2.

**Table 2:** Distribution of the number of falls related to the causes of falls in elderly patients with chronic vestibular dysfunction (n = 64).

	Number of falls		
CAUSES	One fall	Two or more falls	Total
	n (%)	n (%)	n (%)
Tripping	11 (73,3)	4 (26,7)	15 (100,0)
Slipping	11 (84,6)	2 (15,4)	13 (100,0)
Syncope/Darkening of vision	1 (14,3)	6 (85,7)	7 (100,0)
Dizziness/Vertigo	1 (6,3)	15 (93,8)	16 (100,0)
Others	6 (46,2)	7 (53,8)	13 (100,0)
Total	30 (46,9)	34 (53,1)	64 (100,0)

Fisher's exact test

p < 0.001

Corrected Chi-squared test (Yates)

Number of falls and slipping (p = 0.026)

Number of falls and dizziness/vertigo (p = 0.001)

There was no significant association between the variable number of falls and the variables place, time, lighting and direction of fall.

## DISCUSSION

Patients in this study had a relatively high average age (73.62 years), similar to that found by other authors that also consider aging as a possible factor of loss of vestibular function, leading to multiple associated otoneurological symptoms such as vertigo and other forms of dizziness, imbalance, hearing loss, tinnitus, and others^12^, ^13^.

Women were 76.6% of our sample, similar to literature findings. Dizziness is more frequent in women in a 2:114 ratio; women also seem to fall more frequently^5^, ^15^.

A variety of diseases may cause dizziness in the elderly, but metabolic and vascular etiologies are the main causes, due either to primary or secondary injury to the vestibular system^16^, ^17^. Our findings corroborate this conclusion, as we 78.1% of our sample had some circulatory system disease and 54.7% an endocrine nutritional and metabolic disease.

Otoneurological clinical data in our sample was similar to findings in other studies^18^, ^19^. There was a predominance of peripheral vestibular disorders, and the more frequent vestibular conditions. According to Ganança et al.^2^, benign paroxysmal positional vertigo is the most common vestibular disease in the elderly, particularly in females.

Our sample was different from population studies on the elderly in that the rate of recurrent falls was 29.2%, compared to a survey done among the elderly in metropolitan Sao Paulo in which the prevalence of recurrent falls was 11.0%^5^. For Herdman et al.^6^, dizziness of vestibular origin may be a predisposing factor of falls and recurrent falls, as vestibular dysfunction limits control of posture, leading to instability and body misalignment.

In our study, 21.9% of patients had restriction of activities of daily life after the last fall, which is in agreement with Nevitt,4 who found that serious injury or activity limitations reached 25.0% in patients with a history of falls, due to physical damage or fear of falling.

Most falls described by patients with vestibular diseases in our study had occurred out of home in a known place (36.1%). Berg et al.^20^ studied falls in independent elderly people and showed that falls occurred mostly at home in an external environment (35.0%). For the elderly with chronic vestibular dysfunction, tasks seem to become more difficult when the environment requires greater control of posture^21^. Out of home, there is an increased demand on the stabilization of the visual field, head and thoracic movements, and most of all a dynamic balance to face any eventual obstacle^22^.

Falls in patients with vestibular diseases in our study occurred mostly during the morning (51.6%), different from Jekins et al.'s^12^ study, where 52.0% of falls were found to have happened during the afternoon. This may be related to a reduction or disappearance of vestibular signs and/or symptoms mediated by repeated exposure to sensory stimulation as the day elapses^20^.

Half our sample reported falls facing forward (propulsion). The difficulty of elderly with vestibular diseases to use hip reactive strategies in an attempt to control the center of gravity to find a support base in a situation of strong and fast disturbance, could explain most of the falls in the forward direction^23^, ^24^, ^25^. In this approach, the line of vision moves with the position of the head, which might make it more difficult for the elderly with vestibular diseases to stabilize the visual field.

Among the causes of falls in our study patients, vertigo was first (25.0%), followed by tripping (23.4%) and slipping (20.3%). Tripping and slipping were also significant causes in Berg et al.'s^20^ study of the elderly, at 34.0% and 25.0%, respectively. The increased prevalence of vertigo as a cause of falls probably is due to vestibular disease that was present in elderly patients in our study.

We also noticed that the most frequent tasks at the moment of the fall were walking, climbing or going down stairs, activities involving postural transference and bathing. Cohen^22^ observed that for patients aged between 35 and 82 years and with vestibular diseases, raising from a chair was difficult for 94.0%, walking over level surfaces was difficult for 81.0% and bathing and climbing stairs was difficult for 75.0% of these patients. These tasks require good functional body balance systems; when these systems are dysfunctional, such activities become a challenge, compromising control of posture and consequently facilitating falls^21^, ^26^.

Falls are strongly associated with the decline of physical function due to aging; functionally, this means that there is a reduction or loss of the capability to undertake the demands of daily life and its environmental challenges^5^.

Restriction of activities was significantly higher in elderly patients with vestibular diseases that had suffered two or more falls compared to patients that had had only one fall. Restriction of activities may be, at least temporarily, a consequence of trauma, fear of falling, medical orders and/or coexisting diseases^4^, ^27^. Functional motor activities related to cephalic movement balance control may cause dizziness in patients with vestibular diseases, further increasing restriction of activities.

In our study, slipping was the most frequent cause of falls in elderly patients reporting one fall, whereas dizziness and vertigo were more frequent in elderly patients that reported two or more falls during our study period. These findings suggest that elderly patients with vestibular diseases and with a higher susceptibility to body imbalance usually have falls related to vestibular disorders. These findings also underline the importance of fall prevention by control of extrinsic factors such as environmental risk, which may cause slipping.

In line with evidence in literature, Perracini^7^ suggests a number of interventions that may be recommended for dealing with risk factors for falls in the elderly, such as: 1) muscle strengthening programs for the quadriceps and ankle dorsal flexor muscles; 2) balance training to integrate sensory information, to control stability limits, to control thoracic rotation and to increase the effectiveness of movement strategies; 3) prescription and/or adaptation of walking support devices; 4) adaptation of corrective lenses; 5) prescription and adequate use of hearing aids; 6) adequate psychotropic medication; 7) changes in the environment and adaptations as needed done by an occupational therapist; 8) specific physical therapy and pharmacological management of chronic degenerative diseases.

Risk factors for falls should be identified to allow prevention and rehabilitation by specific interventions. We highlight the need for further studies to help identify risk factors for falls in the elderly with vestibular diseases.

## CONCLUSION

The majority of elderly patients that have a diagnosis of chronic vestibular disease report fear of falls and a tendency to fall. Falls are more frequent during the morning, out of home and during walking. Half the patients report the propulsive direction, and the most common causes are vertigo and tripping over something. The number of falls is associated with restriction of activities after the last fall, and with the causes of the fall (slipping and dizziness).
